# Efficacy and safety of omega-3 fatty acids supplementation for anxiety symptoms: a systematic review and dose-response meta-analysis of randomized controlled trials

**DOI:** 10.1186/s12888-024-05881-2

**Published:** 2024-06-18

**Authors:** Negar Bafkar, Sheida Zeraattalab-Motlagh, Ahmad Jayedi, Sakineh Shab-Bidar

**Affiliations:** 1 Department of Community Nutrition, School of Nutritional Science and Dietetics, Tehran University of Medical Sciences, Tehran, Iran; 2 Department of Health and Human Performance, University of Houston, Houston, TX USA; 3 Social Determinants of Health Research Center, Semnan University of Medical Sciences, Semnan, Iran; 4 Sports Medicine Research Center, Neuroscience Institute, Tehran University of Medical Sciences (TUMS), Tehran, Iran

**Keywords:** Omega-3, Anxiety, Meta-analysis, Randomized trials, Supplementation, Mental health

## Abstract

**Background/Objectives:**

There is uncertainty about the optimum dose of omega-3 fatty acids for anxiety symptoms. We aimed to find the dose-dependent effect of omega-3 supplementation on anxiety symptoms.

**Methods:**

We systematically reviewed PubMed, Scopus, and Web of Science until December 2022 to find randomized trials that assessed the effects of omega-3 fatty acids supplementation on anxiety symptoms in adults. Investigators performed the literature search and screened the titles/abstracts and full-texts and between-reviewer agreement was assessed as Cohen’s kappa coefficient. We conducted a random-effects dose-response meta-analysis to estimate standardized mean differences (SMD) and 95% confidence intervals (CIs) and assessed the certainty of evidence using the GRADE framework.

**Results:**

A total of 23 trials with 2189 participants were included. Each 1 gram per day supplementation with omega-3 fatty acids resulted in a moderate decrease in anxiety symptoms (SMD: -0.70, 95%CI: -1.17, -0.22; GRADE = low). The non-linear dose-response analysis indicated the greatest improvement at 2 g/d (SMD: -0.93, 95%CI: -1.85, -0.01), and that supplementation in a dose lower than 2 g/d did not affect anxiety symptoms. Omega-3 fatty acids did not increase adverse events (odds ratio: 1.20, 95%CI: 0.89, 1.61; GRADE = moderate).

**Conclusions:**

The present dose-response meta-analysis suggested evidence of very low certainty that supplementation with omega-3 fatty acids may significantly improve anxiety symptoms, with the greatest improvements at 2 g/d. More trials with better methodological quality are needed to reach more robust evidence.

**Protocol registration:**

PROSPERO (CRD42022309636).

**Supplementary Information:**

The online version contains supplementary material available at 10.1186/s12888-024-05881-2.

## Introduction

Anxiety is a psychological state that arises from excessive or disproportionate fear, and it is the most common psychiatric symptom that can cause distress or impairment [1]. Anxiety disorders are the leading mental disorders in the world [2]. An increase in anxiety symptoms, whether they’re emotional (like fear or apprehension) or physiological (such as a fast heart rate or trembling), is a shared characteristic among these disorders [3]. However, the diagnostic criteria for anxiety disorders vary greatly, including factors like how often and how severe the symptoms are, as well as whether the triggers for these symptoms are specific or broader [3]. In general, cognitive behavioral therapy is the most empirically supported psychological treatment for adults with anxiety disorders [4]. Drug therapies are also available for all anxiety disorders [4]; however, traditional medications, particularly at high doses or long-term usage, have some unfavorable adverse effects, which limit their utilization for the treatment of anxiety disorders [5]. Selective serotonin reuptake inhibitors (SSRIs) are effective in treating anxiety disorders. Furthermore, SNRIs (serotonin-norepinephrine reuptake inhibitors) influence outcomes more than a placebo does. The Food and Drug Administration (FDA) has approved venlafaxine, an SNRI, and the SSRIs paroxetine and sertraline. Benzodiazepines and the beta-blocker propranolol are also used to treat social anxiety disorder. Propranolol has the advantage of being used on an as-needed basis without the risk of developing dependence and tolerance, as exists with benzodiazepines [6].

Nutritional factors have a role in preventing and treating mental disorders [7]. Suboptimal nutrition has been implicated in the pathology of mental disorders and may impede treatment and recovery. Thus, nutritional interventions could potentially treat these disorders and are likely important for prevention [8].

Omega-3 polyunsaturated fatty acids, including α-linolenic acid (ALA) and docosapentaenoic acid (DPA), originate primarily from specific plant sources or are modified in plants, as well as including eicosapentaenoic acid (EPA) and docosahexaenoic acid (DHA) which are almost exclusively found in marine and algal sources [9]. Humans do not efficiently synthesize these fatty acids and need to consume them directly. Marine-derived omega-3 fatty acids (EPA and DHA) regulate dopaminergic and serotonergic neurotransmission and, thus, can affect anxiety symptoms [10]. The central nervous system has the highest concentration of these fatty acids in the human body after adipose tissue [11]. The brain needs sufficient and constant amounts of EPA and DHA for optimum function and a proper structure [12].

The possible mechanisms by which omega-3 related to anxiety were as follows. It is suggested that inflammatory responses are associated with anxiety [13]. Anxiety increases the production of pro-inflammatory cytokines such as interleukin-6 and tumor necrosis factor-alpha [14]. It has been indicated that the consumption of omega-3 reduces the production of pro-inflammatory cytokines [15, 16]. Another possible mechanism is the expression of brain-derived neurotrophic factor (BDNF), which is a protein that can regulate the function of the nervous system [17]. When this protein is low, the synaptic growth of synergistic neurons in the brain is not stimulated, and its insufficient level is associated with depression and anxiety [18, 19]. A review study including six studies with 469 participants showed that the consumption of omega-3 supplements could reduce anxiety symptoms through changes in four major mechanisms including inflammatory response, BDNF, cortisol, and cardiovascular activity [20].

Existing evidence on the efficacy of supplementation with omega-3 fatty acids in reducing anxiety symptoms is insufficient. An intervention trial revealed that omega-3 could improve anxiety among healthy subjects who encountered stressful evaluations [21]. A review study that evaluated the effects of omega-3 on anxiety indicated an improvement in anxiety symptoms (2.1 g/d EPA); however, the number of studies was very low (*n* = 1) [22]. A meta-analysis of intervention studies showed that supplementation with more than 2 g/day did have positive effects on anxiety symptoms [23]. Another review demonstrated that the consumption of omega-3 supplements, particularly through pathways related to inflammation, can lead to a decrease in anxiety symptoms [20]. However, two trials revealed no relation between omega-3 and anxiety disorders [24, 25]. Moreover, the optimum dose of omega-3 fatty acids for reducing anxiety symptoms is still unclear. Therefore, in this systematic review of randomized controlled trials (RCTs), we intended to investigate the dose-dependent effects of omega-3 fatty acid supplementation on anxiety symptoms in adults.

## Methods

We followed the guidelines from the Cochrane Handbook of Systematic Intervention Reviews [26] and the Grading Recommendations, Assessment, Development, and Evaluation (GRADE) handbook to carry out the present systematic study [27]. We registered our protocol for systematic reviews in PROSPERO (CRD42022309636).

### Data sources and searches

We systematically searched three scientific databases, including PubMed, Scopus, and Web of Science, until February 2022, followed by an updated search to December 15, 2022. Working in duplicate, two investigators (NB and SZM) performed the literature search and screened the titles/abstracts and full-text articles in Endnote X9. The between-reviewer agreement was assessed and reported as Cohen’s kappa coefficient (κ) [28]. Disagreements were resolved by discussion with a third reviewer (SS-B). We also reviewed the reference list of meta-analysis studies of RCTs that investigated the effect of omega-3 on anxiety symptoms. Our search is limited to English-language articles. We described the complete search strategy in Table 1.

**Table 1:** Search strategy (PubMed) to find potential eligible trials for inclusion in a dose-response meta-analysis of omega-3 fatty acids supplementation on anxiety symptoms (2020/12/15)

1. omega-3[tiab] OR *n*-3[tiab] OR “omega-3 fatty acid”[tiab] OR “ω-3 fatty acid”[tiab] OR “*n*-3 fatty acid”[tiab] OR “fish oil”[tiab] OR lipids[tiab] OR “ω-3 FA”[tiab] OR “polyunsaturated fatty acids”[tiab] OR w-3[tiab] OR EPA[tiab] OR DHA[tiab] OR ALA[tiab] OR eicosapentaenoic[tiab] OR docosahexaenoic[tiab] OR “alpha-linolenic acid”[tiab] OR “marine oil”[tiab] OR “long chain polyunsaturated fatty acids”[tiab] OR prostaglandins[tiab] “*N*-3 polyunsaturated fatty acids”[tiab] OR PUFAs[tiab] OR “*n*-3 PUFA”[tiab] OR “α-Linolenic acid”[tiab] OR “Fatty Acids, Omega-3“[Mesh] OR “Fish Oils“[Mesh] OR “Eicosapentaenoic Acid“[Mesh] OR “Docosahexaenoic Acids“[Mesh] OR “Prostaglandins“[Mesh]	
2. (Depress[tiab] OR “affective disorder”[tiab] “Phobic Disorders“[tiab] OR “affective illness”[tiab] OR “mood disorder”[tiab] OR internalizing[tiab] OR “mental health”[tiab] OR “mental illness”[tiab] OR “psychiatric disorder”[tiab] OR “psychiatric illness”[tiab] OR Depression[tiab] OR Anxiety[tiab] OR “Depressive Disorder”[tiab] OR depressive OR “anxiety disorders”[tiab] OR depression[tiab] OR panic[tiab] or phobia[tiab] OR “Mood Disorders“[Mesh] OR “Mental Health“[Mesh] OR “Mental Disorders“[Mesh] OR “Depression“[Mesh] OR “Anxiety“[Mesh] OR “Panic“[Mesh] OR “Phobic Disorders“[Mesh])	
3. intervention[tiab] OR RCT[tiab] OR “controlled trial“[tiab] OR randomized[tiab] OR random[tiab] OR Randomly[tiab] OR Placebo[tiab] OR Assignment[tiab] OR “clinical trial“[tiab] OR trial[tiab] OR randomised[tiab] OR “Methods“[Mesh] OR “Randomized Controlled Trial“[Publication Type] OR “Controlled Clinical Trial“[Publication Type] OR “Placebos“[Mesh] OR “Placebo Effect“[Mesh] OR “Clinical Trial“[Publication Type] OR “Clinical Trials as Topic“[Mesh]	
14. 1 AND 2 AND 3	

### Study selection

The inclusion criteria for the present review were determined according to the PICOS (population, intervention/exposure, comparator, outcome, as well as study design) approach and included the following items: (1) RCTs (study design), conducted on adults over 18 years of age, independent of drug usage or health status (population); (2) evaluation of the effect of oral omega-3 supplements including EPA, DHA, or ALA, in combination or individually and in various forms such as pills, oils, or fortified foods (intervention), compared to a control group (comparator); (3) considered a change in anxiety symptoms, assessed by formal diagnosis or an appropriate scale as continuous scale in participants with or without existing anxiety, as an outcome; (4) provided mean and standard deviation (SD) of anxiety symptoms at baseline and end of the study or reported sufficient information to estimate these values; and (5) provided dose of omega-3 supplementation in the intervention group. On the other hand, RCTs conducted on individuals under 18 years old, including pregnant or lactating women, were excluded.

### Outcomes

Our primary outcome was a change in anxiety symptoms, while secondary outcomes were adverse events and health-related quality of life (HRQoL) and its components, including physical components, pain, general health, emotional well-being, and social functioning [29].

### Data extraction

After the screening of the full texts, two investigators (NB and SZM) independently and in duplicate extracted the following characteristics from each trial: author’s last name, publication year, country, age range, baseline body mass index (BMI), sex, total sample size, duration of intervention, type intervention characteristics (dose of omega-3 supplementation in the intervention group), comparison group, calorie restriction, anxiety scale, baseline anxiety, any antidepressant drug usage, health status, outcome measures and main results for the outcomes included.

### Risk of bias assessment

We evaluated the risk of bias using version 2.0 of the Cochrane tool for risk of bias [30]. Two authors (NB and SZM) independently evaluated the risk of bias in the trials, with disagreements resolved by the third author (AJ) when necessary.

### Data synthesis and analysis

We considered the standardized mean difference (SMD) and its 95% confidence interval (CI) of changes in anxiety symptoms in the intervention group compared to the control group as the effect size for reporting the results of the present systematic review. First, we extracted the mean and SD of changes from baseline till the end of the intervention in each study arm in each trial. For those trials that did not report these changes, we calculated these values using the reported means and SDs of outcomes before and after the intervention using the Cochrane Handbook guidelines [26]. In the case of trials that reported standard errors instead of SDs, we converted them to SDs [31]. If SDs or standard errors were not reported in the trials, we used the mean SDs received from other trials for the analyses [32]. Second, for the analyses of continuous outcomes, we calculated SMD and its 95%CI for each 1 g/d increase in omega-3 fatty acids intake in each RCT using the approach introduced by Crippa and Orsini [33]. This method needs the number of participants in each study arm, dose of intervention, and the mean and SD of change across the study arm in each trial. Trial-specific mean and standard error of changes in outcomes for each 1 g/d increase in omega-3 fatty acids intake were pooled by applying the DerSimonian and Laird random-effects model [34]. We used SMD as an effect estimate because intervention trials used different questionnaires or scales (including the Barratt Impulsiveness Scale or Behavioral Inhibition/Behavioral Activation scale, etc.) to assess anxiety symptoms.

We performed predefined subgroup analyses according to baseline anxiety risk (high risk, specified as individuals with clinically diagnosed anxiety, using any diagnostic criteria; medium risk, specified as individuals with anxiety risk factors, such as long-term conditions; and low risk, specified as all other populations), intervention duration (≤ 12 versus ≥ 12 weeks) and risk of bias (high risk of bias, low risk of bias, some concerns). We selected baseline anxiety risk since it is an important effect modifier according to previous research. We also selected the risk of bias according to the GRADE instructions to determine whether the effects can also be seen in high-quality trials. Since adherence to the dietary interventions reduces over intervention duration, we selected intervention duration to determine whether the effects persisted in the long term. We also performed a meta-regression analysis to test the effect of intervention duration as a potential effect modifier.

Moreover, post-hoc subgroup analyses were according to the variables found in the literature search including supplement type (EPA, DHA, EPA + DHA), sex (men, women, both), weight status (normal weight, overweight/obese, not reported), health status (depressed, individuals with substance use, stressed, healthy, self-harm experience, ischemic stroke, Alzheimer disease, premenstrual syndrome), and antidepressant drugs usage (yes, no, mixed, not reported). According to eight criteria determined by the Instrument to assess the Credibility of Effect Modification Analyses (ICEMAN), we investigated the credibility of subgroup differences when the *p*-value for subgroup difference was < 0.10 [32]. ICEMAN consisted of 8 criteria to assess the credibility of the observed subgroup effects, one of which is the *p*-value for subgroup difference. According to the ICEMAN, when p for subgroup difference is 0.01–0.05, chance is a likely explanation, and when *P* < 0.01, chance is an unlikely explanation. We followed their advanced approach to avoid over interpretation of subgroup effects (Supplementary Table 2).

We applied meta-regression analysis to calculate the *p*-value for subgroup differences. We examined the potential influence of any trial on the primary results by applying influence analysis and removing any RCT at once. We applied Egger’s [35] and Begg’s [36] tests for publication bias and examined asymmetry in the funnel plots. For assessing the heterogeneity across trials, we applied the I^2^ statistic and conducted a χ2 test (P_heterogeneity_>0.10) [37]. Finally, we did a dose-response meta-analysis to clarify the dose-dependent effects of omega-3 fatty acids (g/d) on anxiety symptoms [38]. For the analyses of binary outcomes (adverse events), we computed the odds ratio and risk difference and their 95%CI using the number of participants and events in the intervention and control groups. STATA software version 17.0 was used for the analyses. A two-tailed *p*-value < 0.05 was regarded as statistically significant.

### Grading of the evidence

We used the GRADE method to evaluate the certainty of evidence [39]. According to the GRADE approach, evidence obtained from RCTs is of high certainty, which can be downgraded or upgraded by predetermined criteria. To the interpretation of the magnitude of effect sizes, the estimated SMDs were interpreted as a trivial and unimportant effect (0.0-0.2), a small effect (0.2–0.6), a moderate effect (0.6–1.2), a large effect (1.2-2.0), a very large effect (2.0–4.0), and an extremely large effect (≥ 4.0) [40, 41].

## Results

### Systematic search

**Fig. 1:**
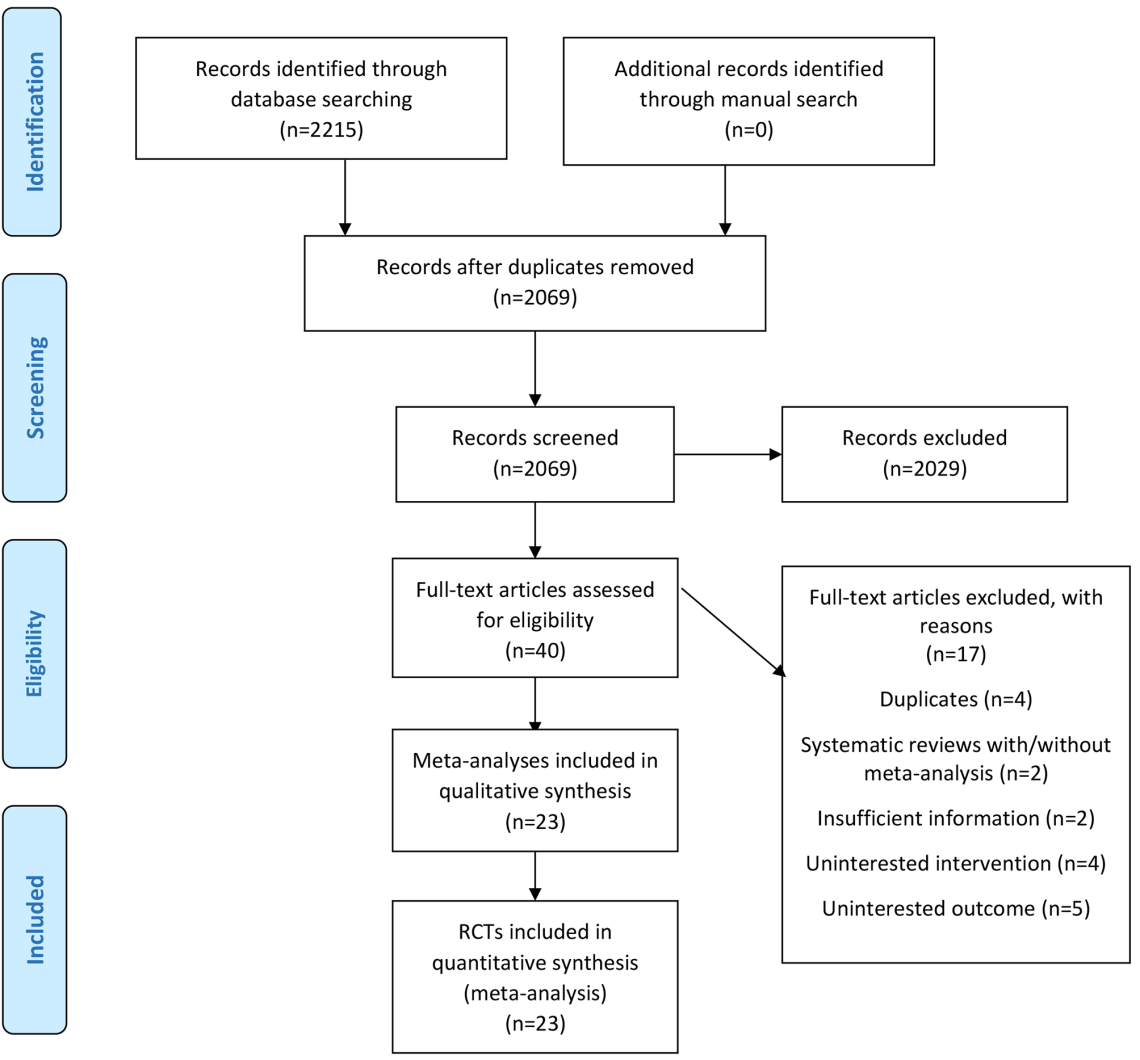
Literature search and study selection process

Figure 1 shows that the database and reference list searches identified 2215 records. After excluding 146 duplicates and an additional 2029 records through screening the title and abstract, 40 full texts were assessed for eligibility. Overall, 23 trials with 2,189 participants were eligible for inclusion in this dose-response meta-analysis [21, 42–62]. The between-reviewer agreement for including studies was near perfect (Cohen’s kappa = 0.85) at the full-text screening step. The list of excluded full-text studies is shown in Supplementary Table 3.

### Characteristics of original trials

The general characteristics of the trials included in the present dose-response meta-analysis are described in Supplementary Table 4. Eligible trials were published between 2007 and 2020. In terms of health status, the subjects in the seven trials were depressed [42, 46, 47, 53, 54, 58, 61], three trials included those with Parkinson’s disease [55, 56, 59], and one trial included participants with premenstrual syndrome [60], individuals with substance use [45], acute myocardial infarction [50], those with self-harm experience [51], stress [44], and stroke [57]. The other seven trials were conducted in healthy individuals. Of the 23 trials, eight were conducted in participants with normal weight [42, 43, 45, 51, 52, 55, 59, 60], seven trials were conducted in those with overweight [44, 45, 48, 53, 55, 57, 62], and three in those with obesity [46, 47, 54]. Seven trials did not report the weight status of the participants in the study [21, 49, 50, 56, 58, 61, 63]. Nineteen out of the 23 trials had an intervention period of 12 weeks or less [21, 42–55, 57, 58, 60, 61], and the other four trials had an intervention duration longer than 12 weeks [56, 59, 62, 63]. Of the trials, 21 studies implemented a combination of EPA and DHA supplements [21, 42–46, 48–55, 57–63], one study implemented DHA supplementation [56], and one trial implemented EPA supplementation [47]. Of 23 trials, seven trials had a low risk of bias (31, 33, 43, 45, 46, 49, 52), four had some concerns [49, 53, 57, 63], and twelve were rated to have a high risk of bias (32, 34–37, 39–41, 44, 48, 50, 51) (Supplementary Table 5).

### Primary outcome

Twenty-three trials with 1093 participants in the intervention group and 1096 in the control group reported information about the effect of omega-3 supplementation on anxiety symptoms [21, 42–63]. Each 1 gram per day of omega-3 fatty acids resulted in a moderate decrease in anxiety symptoms (SMD: -0.70, 95% CI: -1.17, -0.22, *p* < 0.001; I^2^ = 97%; P_heterogeneity_ < 0.001, GRADE = very low, Table 2) (Supplementary Fig. 1).

**Table 2:** The effect of omega-3 fatty acids on primary and secondary outcomes

Outcome (s)	Number of trials (participants)	Type of effect size	Effect size (95%CI)	*P*-value for the effect	I^2^, *P*_heterogeneity_	Tau^2^	GRADE certainty
Anxiety symptoms (per 1 g/d)	23 (2189)	Standardized mean difference	-0.70 (-1.17, -0.22)	< 0.001	97%, < 0.001	0.9838	Low
Adverse events	8 (1161)	Odds ratio Risk difference	1.20 (0.89, 1.61) 0.06 (-0.02, 0.13)	0.23	46%, 0.07	0.1797	Moderate
Emotional well-being	1 (72)	Standardized mean difference	-0.23 (-0.69, 0.23)	0.32	-	-	Very low
General health	1 (72)	Standardized mean difference	-0.25 (-0.71, 0.21)	0.29	-	-	Very low
Pain	1 (72)	Standardized mean difference	-0.33 (-0.79, 0.13)	0.16	-	-	Very low
Physical component scale	2 (174)	Standardized mean difference	-0.19 (-0.48, 0.11)	0.21	0, 0.69	0.0000	Very low
Social functioning	1 (72)	Standardized mean difference	0.52 (0.05, 0.98)	0.03	-	-	Very low

Table 3 indicates the subgroup analyses of the effects of omega-3 fatty acids (each 1 g/d) on anxiety symptoms. There was a significant subgroup difference by study risk of bias, where trials with a high risk of bias indicated a large and significant effect, but those with a low risk of bias did not indicate significance, as well as the magnitude of the findings. There were also other significant subgroup differences by weight status, medication use, and baseline risk of anxiety; however, in those cases, chance was a likely explanation (p subgroup difference between 0.01 and 0.05), and the credibility of subgroup difference was rated low (Supplementary Table 2) [32]. We did not find a significant or credible difference by intervention duration which was confirmed by meta-regression analysis (SMD per one-week increase: -0.01, 95%CI: -0.31, 0.06; *P* = 0.54).

**Table 3:** Subgroup analysis of the effects of omega-3 fatty acids (each 1 g/d) on anxiety symptoms

	*n*	Standardized mean difference (95%CI)	*P* for the effect	I^2^, *P*_heterogeneity_	*P* subgroup difference ^1^
All trials	23	-0.70 (-1.17, -0.22)	< 0.001	97%, < 0.001	-
Risk of bias					0.01
Low	7	0.07 (-0.41, 0.55)	0.78	58%, 0.02	
Some concerns	4	-0.27 (-0.86, 0.32)	0.64	47%, 0.13	
High	12	-1.12 (-1.75, -0.48)	< 0.001	98%, < 0.001	
Intervention duration					0.45
≤ 12 weeks	19	-0.71 (-1.23, -0.18)	< 0.001	97%, < 0.001	
> 12 Weeks	4	-0.36 (-1.08, 0.35)	0.37	78%, < 0.001	
Supplement type					0.08
DHA	1	-2.00 (-6.24, 2.24)	0.81	-	
EPA	1	-0.45 (-1.52, 0.42)	0.24	-	
EPA + DHA	17	-0.75 (-1.27, -0.23)	< 0.001	97%, < 0.001	
Sex					0.61
Men	-	-	-	-	
Women	4	-0.47 (-1.35, 0.40)	0.61	95%, < 0.001	
Both	19	-0.76 (-1.43, -0.09)	0.002	97%, < 0.001	
Weight status					0.01
Normal weight	6	-0.19 (-1.05, 0.67)	0.41	97%, < 0.001	
Overweight/obese	10	-0.09 (-0.48, 0.67)	0.58	90%, < 0.001	
Not reported	7	-2.70 (-4.40, -0.99)	< 0.001	98%, < 0.001	
Health status					0.001
Depressed	7	-1.16 (-2.98, 0.67)	0.74	98%, < 0.001	
Individuals with substance use	1	-0.84 (-0.95, -0.73)	< 0.001	-	
Stressed	1	-0.45 (-1.30, 0.40)	0.36	-	
Healthy	7	-0.10 (-0.40, 0.20)	0.28	73%, 0.001	
Post myocardial infarction	1	-5.20 (-7.47, -2.93)	0.001	-	
Self-harm experience	1	2.69 (0.43, 4.95)	0.01	-	
Parkinson disease	3	-1.93 (-4.30, 0.44)	0.37	0%, 0.49	
Ischemic stroke	1	0.71 (-0.39, 1.80)	0.19	-	
Premenstrual syndrome	1	-1.21 (-1.31, -1.11)	< 0.001	-	
Antidepressant drugs usage					0.02
Yes	9	-1.13 (-1.89, -0.44)	< 0.001	97%, < 0.001	
No	13	-0.17 (-0.64, 0.30)	0.43	79%, 0.001	
Mixed	1	-3.68 (-7.76, 0.41)	0.21	-	
Baseline risk of anxiety					0.02
Low	7	-0.11 (-0.42, 0.20)	0.24	74% ,0.001	
Medium	5	-0.76 (-1.14, -0.39)	< 0.001	89%, < 0.001	
High	11	-1.27 (-2.80, 0.27)	0.20	98%, < 0.001	

^1^ Obtained by metaregression analysis.

Abbreviations: ALA, α-linolenic acid; DHA, docosapentaenoic acid; EPA, eicosapentaenoic acid.

**Fig. 2:**
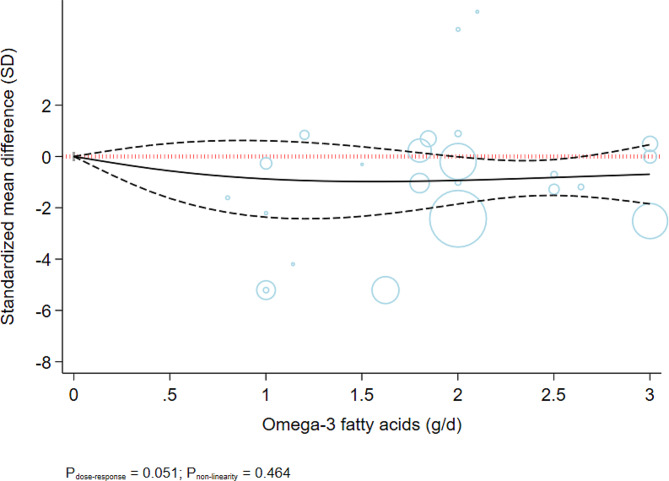
Dose-dependent effect of omega-3 fatty acids on anxiety symptoms. Solid lines represent standardized mean difference and dashed lines represent 95% confidence interval

We observed no indication of publication bias with Egger’s test (*P* = 0.61), Begg’s test (*P* = 0.13), or with the inspection of the funnel plot (Supplementary Fig. 2). The dose-dependent effects of omega-3 on anxiety symptoms are shown in Table 4; Fig. 2. The non-linear dose-response analysis indicated the greatest improvement at 2 g/d, where we found a moderate improvement in anxiety symptoms (SMD_2g/d_: -0.93; 95%CI: -1.85, -0.01) (P_dose−response_: 0.051, P_nonlinearity_ = 0.464; *n* = 23, Fig. 2).

**Table 4:** The effects of omega-3 fatty acids on anxiety symptoms from the nonlinear dose-response meta-analysis (standardized mean difference and 95% confidence interval)

Omega-3 fatty acids supplement	0 (ref)	0.5 g/day	1 g/day	1.5 g/day	2 g/day	2.5 g/day	3 g/day
Anxiety symptoms	0	-0.56 (-1.64, 0.51)	-0.88 (-2.37, 0.61)	-0.98 (-2.33, 0.38)	-0.93 (-1.85, -0.01)	-0.82 (-1.52, -0.12)	-0.69 (-1.85, 0.46)

### Secondary outcomes

The effect of omega-3 fatty acids on secondary outcomes is reported in Table 2. Omega-3 fatty acids did not increase adverse events. Supplementation with omega-3 fatty acids resulted in a small increase in social functioning (SMD: 0.52, 95% CI: 0.05, 0.98; GRADE = very low), but it did not increase other aspects of quality of life such as emotional well-being, general health, and physical component scale.

### Grading of the evidence

The certainty of evidence was rated very low for the effects of omega-3 fatty acids on anxiety symptoms. It was rated moderate for the effects of supplementation with omega-3 fatty acids on adverse events. The certainty of evidence was rated very low for other outcomes (Supplementary Table 6).

## Discussion

The present meta-analysis of intervention trials was the first study that addresses previous review limitations. It examines the dose-dependent effect of omega-3 fatty acids on anxiety symptoms, applying methodologies not previously utilized in similar studies. These methodologies include dose-dependent analysis to determine the optimal dosage for improving anxiety symptoms, utilization of ICEMAN for evaluating the credibility of subgroup differences, and application of the GRADE approach to assess the certainty of evidence in the included studies. We also conducted subgroup analysis according to baseline risk of anxiety, health status, and any antidepressant drug usage to identify which group benefits the most from omega-3 supplementation. Our findings showed that each 1 g/d omega-3 could moderately reduce anxiety symptoms. The dose-response meta-analysis suggested the greatest improvement at 2 g/d, and that higher doses of omega-3 supplements did not confer added health benefits.

A previous meta-analysis of 19 clinical trial articles (*n* = 2240 participants) demonstrated that omega-3 consumption in a dose below 2 g per day did not show any significant effects on anxiety symptoms. The participants included in the studies summarized in this meta-analysis were both healthy individuals and individuals with either a physical illness or mental disorder [23]. Another review study among individuals with no serious illness, including 31 trials, indicated that increasing omega-3 intake (300–3360 mg/d) might have little or no effect on reducing anxiety symptoms [64]. A reason for the contradictory results may be that in most original studies, the dose of omega-3 consumed was less than 2 g. Our dose-response meta-analysis indicated that supplementation with omega-3 fatty acids at a dose lower than 2 g/d did not significantly reduce anxiety symptoms.

Moreover, the subgroup analysis failed to show a significant and credible subgroup difference by intervention duration, and only four trials had an intervention duration longer than 12 weeks. In addition, we found a significant subgroup difference by study risk of bias, where trials with a low risk of bias did not show a significant effect. Therefore, more trials with better methodological quality and longer intervention duration are needed in this field.

We did not find a significant subgroup difference by supplement type (EPA versus DHA versus combined). This might be because the number of included studies for EPA and DHA supplements was very low (for EPA (*n* = 1) and DHA (*n* = 1); as a result, we were unable to find the difference between EPA and DHA. An intervention trial study indicated a significant decrease in anxiety and angry symptoms by daily intake of EPA & DHA (2,250 mg/d of EPA + 500 mg/d of DHA) for 12 weeks. They also indicated that both EPA and DHA serum levels were incremented; however, the EPA was more effective in improving anxiety symptoms and DHA was more effective in improving anger symptoms. This might be because of the different modes of action of long-chain omega-3 fatty acids for anger and anxiety [45]. Administration of EPA could reduce anxiety behavior in rats, as well as the stimulation of corticosterone by interleukin-1 beta [65]. Besides, changes in the way serotonin (5-HT) neurotransmission works in the brain can contribute to violent behavior, and consuming more DHA could help increase 5-HT neurotransmission in the brain. A study has shown that people with higher levels of plasma DHA were more likely to have higher levels of cerebrospinal fluid 5-Hydroxyindole Acetic Acid for both healthy individuals and late-onset alcoholics [66].

A cross-sectional study of 935 Australian adults indicated that those in the upper quartile of DHA intake had half of anxiety disorders compared to those in the lower quartile of DHA intake. No significant relationship was found for other types of omega-3 fatty acids, such as EPA [67]. However, of the 23 trials included in the present review, 21 used a combination of EPA and DHA, and thus, more trials are needed to determine whether EPA or DHA are superior to each other in reducing anxiety symptoms.

Regarding any antidepressant drug usage, we found a significant subgroup difference, where supplementation with omega-3 could significantly reduce the risk of anxiety symptoms in individuals who used any antidepressant drugs compared to individuals who did not use any antidepressant drugs or in combination. These findings indicate that omega-3 fatty acids might be more effective in individuals who use antidepressant drugs. A research study has shown that incorporating omega-3 as an add-on therapy has significantly enhanced the clinical effectiveness of antidepressant drugs such as sertraline. For example, combining antidepressant drugs with dietary and physiological supplements has amplified their antidepressant effects [68, 69].

Our dose-response meta-analysis suggested that supplementation with omega-3 fatty acids at a dose of lower than 2 g/d had no effects on anxiety symptoms in adults. The greatest impact was also seen at this dose. This was consistent with a previous pairwise meta-analysis of intervention studies that suggested that the effects of omega-3 fatty acids on anxiety symptoms were stronger in the subgroup of trials with higher doses (at least 2 g/d) [23]. Our results provided additional practical information, suggesting that supplementation at a dose higher than 2 g/d did not confer additional decrement in anxiety symptoms.

### Strengths and limitations

Our review had several strengths. First, this review was the first study to examine the dose-dependent effect of omega-3 fatty acids on anxiety symptoms. Second, we rated the certainty of evidence using the GRADE approach and utilized the MCID thresholds to determine whether the results were clinically important. Lastly, we conducted a subgroup analysis to find the source of heterogeneity and used the recently released ICEMAN tool for subgroup analyses. Among the limitations of our study, the variety of assessment methods for anxiety symptoms may also limit clinical interpretation and generalizability of the results. Moreover, we did not evaluate the potential effect modification by baseline omega-3 status in the study participants, which may affect our results. Examining this issue can help determine if supplementation is only required for deficient individuals or if it’s effective in all individuals, including those with normal intake. Also, we included adults regardless of their anxiety and health status. The results of our subgroup analysis also showed no significant effects in the subgroup of individuals with depression and high risk (baseline anxiety risk). Therefore, researchers should be careful about the interpretation of the results and discuss more carefully about these findings. Considering these limitations, more trials should be done on the possible effects of omega-3 on anxiety in individuals with anxiety and depression. Finally, we included only four studies with long-term duration, and thus, we could not thoroughly investigate the long-term effect of omega-3 fatty acids on anxiety symptoms.

## Conclusions

In this systematic review and meta-analysis, it was seen that the consumption of each 1 gram of omega-3 per day reduced anxiety symptoms, but certainty of evidence was rated low. Dose-dependent analysis revealed that the maximum reduction in anxiety symptoms was seen in a dose of 2 g/d. However, further controlled trials with long-term follow-up and considering the baseline omega-3 status of the participants, as well as possible effects of omega-3 on individuals with anxiety and depression are needed to indicate more accurate results.

### Electronic supplementary material

Below is the link to the electronic supplementary material.

Supplementary Materials

## Data Availability

The data sets generated or analyzed during the current study are not publicly available but are available from the corresponding author at reasonable request.

## Declarations

### Transparency declaration

The lead author affirms that the manuscript is an honest, accurate, and transparent account of the studies being reported; that no important aspects of the study have been omitted; and that any discrepancies from the study as planned (and, if relevant, registered) have been explained.
