# Supplementary material for: Efficacy and safety of omega-3 fatty acids supplementation for anxiety symptoms: a systematic review and dose-response meta-analysis of randomized controlled trials
Source: BMC Psychiatry. 2024 Jun 18;24:455. doi: 10.1186/s12888-024-05881-2 (PMC11186166; doi:10.1186/s12888-024-05881-2)
Supplement: Supplementary file 1 — Supplementary Materials [file 12888_2024_5881_MOESM1_ESM.doc]

**The effect of omega-3 supplementation for anxiety symptoms: a systematic review and dose-response meta-analysis of randomized controlled trials**

Supplementary Tables S1-S6 and Supplementary Figures S1-S4

**Supplementary Table 1.** PRISMA checklist 2020.

| **Section and Topic** | **Item #** | **Checklist item** | **Location where item is reported** |
| --- | --- | --- | --- |
| **TITLE** | | |  |
| Title | 1 | Identify the report as a systematic review. | 1 |
| **ABSTRACT** | | |  |
| Abstract | 2 | See the PRISMA 2020 for Abstracts checklist. | 2 |
| **INTRODUCTION** | | |  |
| Rationale | 3 | Describe the rationale for the review in the context of existing knowledge. | 4 |
| Objectives | 4 | Provide an explicit statement of the objective(s) or question(s) the review addresses. | 4 |
| **METHODS** | | |  |
| Eligibility criteria | 5 | Specify the inclusion and exclusion criteria for the review and how studies were grouped for the syntheses. | 5 |
| Information sources | 6 | Specify all databases, registers, websites, organisations, reference lists and other sources searched or consulted to identify studies. Specify the date when each source was last searched or consulted. | 4-5 |
| Search strategy | 7 | Present the full search strategies for all databases, registers and websites, including any filters and limits used. | Table 1 |
| Selection process | 8 | Specify the methods used to decide whether a study met the inclusion criteria of the review, including how many reviewers screened each record and each report retrieved, whether they worked independently, and if applicable, details of automation tools used in the process. | 5 |
| Data collection process | 9 | Specify the methods used to collect data from reports, including how many reviewers collected data from each report, whether they worked independently, any processes for obtaining or confirming data from study investigators, and if applicable, details of automation tools used in the process. | 5 |
| Data items | 10a | List and define all outcomes for which data were sought. Specify whether all results that were compatible with each outcome domain in each study were sought (e.g. for all measures, time points, analyses), and if not, the methods used to decide which results to collect. | 6 |
|  | 10b | List and define all other variables for which data were sought (e.g. participant and intervention characteristics, funding sources). Describe any assumptions made about any missing or unclear information. | 6 |
| Study risk of bias assessment | 11 | Specify the methods used to assess risk of bias in the included studies, including details of the tool(s) used, how many reviewers assessed each study and whether they worked independently, and if applicable, details of automation tools used in the process. | 6 |
| Effect measures | 12 | Specify for each outcome the effect measure(s) (e.g. risk ratio, mean difference) used in the synthesis or presentation of results. | 6-7 |
| Synthesis methods | 13a | Describe the processes used to decide which studies were eligible for each synthesis (e.g. tabulating the study intervention characteristics and comparing against the planned groups for each synthesis (item #5)). | 6 |
|  | 13b | Describe any methods required to prepare the data for presentation or synthesis, such as handling of missing summary statistics, or data conversions. | 7-8 |
|  | 13c | Describe any methods used to tabulate or visually display results of individual studies and syntheses. | 7-8 |
|  | 13d | Describe any methods used to synthesize results and provide a rationale for the choice(s). If meta-analysis was performed, describe the model(s), method(s) to identify the presence and extent of statistical heterogeneity, and software package(s) used. | 7-8 |
|  | 13e | Describe any methods used to explore possible causes of heterogeneity among study results (e.g. subgroup analysis, meta-regression). | 7-8 |
|  | 13f | Describe any sensitivity analyses conducted to assess robustness of the synthesized results. | NA |
| Reporting bias assessment | 14 | Describe any methods used to assess risk of bias due to missing results in a synthesis (arising from reporting biases). | 6-7 |
| Certainty assessment | 15 | Describe any methods used to assess certainty (or confidence) in the body of evidence for an outcome. | 7-8 |
| **RESULTS** | | |  |
| Study selection | 16a | Describe the results of the search and selection process, from the number of records identified in the search to the number of studies included in the review, ideally using a flow diagram. | 9 |
|  | 16b | Cite studies that might appear to meet the inclusion criteria, but which were excluded, and explain why they were excluded. | Table S2 |
| Study characteristics | 17 | Cite each included study and present its characteristics. | Table S3 |
| Risk of bias in studies | 18 | Present assessments of risk of bias for each included study. | Table S4 |
| Results of individual studies | 19 | For all outcomes, present, for each study: (a) summary statistics for each group (where appropriate) and (b) an effect estimate and its precision (e.g. confidence/credible interval), ideally using structured tables or plots. | Table 2 |
| Results of syntheses | 20a | For each synthesis, briefly summarise the characteristics and risk of bias among contributing studies. | 9-11 |
|  | 20b | Present results of all statistical syntheses conducted. If meta-analysis was done, present for each the summary estimate and its precision (e.g. confidence/credible interval) and measures of statistical heterogeneity. If comparing groups, describe the direction of the effect. | 10-11 |
|  | 20c | Present results of all investigations of possible causes of heterogeneity among study results. | 9-11 |
|  | 20d | Present results of all sensitivity analyses conducted to assess the robustness of the synthesized results. | NA |
| Reporting biases | 21 | Present assessments of risk of bias due to missing results (arising from reporting biases) for each synthesis assessed. | NA |
| Certainty of evidence | 22 | Present assessments of certainty (or confidence) in the body of evidence for each outcome assessed. | 11 |
| **DISCUSSION** | | |  |
| Discussion | 23a | Provide a general interpretation of the results in the context of other evidence. | 11-16 |
|  | 23b | Discuss any limitations of the evidence included in the review. | 14-15 |
|  | 23c | Discuss any limitations of the review processes used. | 14-15 |
|  | 23d | Discuss implications of the results for practice, policy, and future research. | 14-16 |
| **OTHER INFORMATION** | | |  |
| Registration and protocol | 24a | Provide registration information for the review, including register name and registration number, or state that the review was not registered. | 4 |
|  | 24b | Indicate where the review protocol can be accessed, or state that a protocol was not prepared. | 4 |
|  | 24c | Describe and explain any amendments to information provided at registration or in the protocol. | NA |
| Support | 25 | Describe sources of financial or non-financial support for the review, and the role of the funders or sponsors in the review. | 16 |
| Competing interests | 26 | Declare any competing interests of review authors. | 16 |
| Availability of data, code and other materials | 27 | Report which of the following are publicly available and where they can be found: template data collection forms; data extracted from included studies; data used for all analyses; analytic code; any other materials used in the review. | 16 |

*From:*  Page MJ, McKenzie JE, Bossuyt PM, Boutron I, Hoffmann TC, Mulrow CD, et al. The PRISMA 2020 statement: an updated guideline for reporting systematic reviews. BMJ 2021;372:n71. doi: 10.1136/bmj.n71

For more information, visit: <http://www.prisma-statement.org/>

**Supplementary Table 2.** Assessment of credibility of subgroup difference for the effects of omega-3 fatty acids on anxiety symptoms based on ICEMAN.

| Variable | Q1 | Q2 | Q3 | Q4 | Q5 | Q6 | Q7 | Q8 | Overall credibility |
| --- | --- | --- | --- | --- | --- | --- | --- | --- | --- |
| Risk of bias | Completely between | Probably not similar | Rather small | Definitely yes | Chance a likely explanation | Definitely yes | Definitely yes | not applicable | Low |
| Supplement type | Completely between | Probably not similar | Very small | Definitely yes | Chance a very likely explanation | Definitely yes | Definitely yes | not applicable | Low |
| Weight status | Completely between | Probably not similar | Rather small | Definitely yes | Chance a likely explanation | Definitely no | Definitely yes | not applicable | Low |
| Depression status | Completely between | Probably not similar | Very small | Definitely no | Chance an unlikely explanation | Definitely no | Definitely yes | not applicable | Low |
| Medication use | Completely between | Probably not similar | Very small | Definitely no | Chance a likely explanation | Definitely no | Definitely yes | not applicable | Low |
| Baseline risk of anxiety | Completely between | Probably not similar | Rather large | Definitely yes | Chance a likely explanation | Definitely yes | Definitely yes | not applicable | Low |

Q, question; Q1, Is the analysis of effect modification based on comparison within rather than between trials? Q2, For within-trial comparisons, is the effect modification similar from trial to trial? Q3, For between-trial comparisons, is the number of trials large? Q4, Was the direction of the effect modification correctly hypothesized priori? Q5, Does a test for interaction suggest that chance is an unlikely explanation of the apparent effect modification? Q6, Did the authors test only a small number of effect modifiers? Q7, Did the authors use a random effects model? Q8, If the effect modifier is a continuous variable, were arbitrary cut points avoided?

**Supplementary Table 3.** List of excluded studies and reasons (n= 17).

| Study | Year | Title | Reason for exclusion |
| --- | --- | --- | --- |
| Andrieu | 2017 | Effect of long-term omega 3 polyunsaturated fatty acid supplementation with or without multidomain intervention on cognitive function in elderly adults with memory complaints (MAPT): a randomised, placebo-controlled trial | Not interested outcome |
| Antypa | 2009 | Omega-3 fatty acids (fish-oil) and depression-related cognition in healthy volunteers | Not sufficient information |
| Assisi | 2006 | Fish oil and mental health: the role of n-3 long-chain polyunsaturated fatty acids in cognitive development and neurological disorders | Systematic review |
| Bellino | 2014 | Efficacy of omega-3 fatty acids in the treatment of borderline personality disorder: A study of the association with valproic acid | Not interested intervention |
| Bot | 2010 | Supplementation with Eicosapentaenoic Omega-3 Fatty Acid Does Not Influence Serum Brain-Derived Neurotrophic Factor in Diabetes Mellitus Patients with Major Depression: A Randomized Controlled Pilot Study | Duplicate |
| Ciappolino | 2017 | The role of n-3 polyunsaturated fatty acids (n-3PUFAs) in affective disorders | Systematic review |
| Estruch | 2018 | Retraction and republication: Primary prevention of cardiovascular disease with a Mediterranean diet. N Engl J Med 2013; 368:1279-90. N Engl J Med. 2018; 378: 25-. | Not interested intervention |
| Fourrier | 2020 | Rapeseed oil fortified with micronutrients improves cognitive alterations associated with metabolic syndrome | Not interested intervention |
| Karr | 2012 | Omega-3 Polyunsaturated Fatty Acids and Cognition in a College-Aged Population | Not sufficient information |
| Kuszewski | 2020 | An Exploratory Analysis of Changes in Mental Wellbeing Following Curcumin and Fish Oil Supplementation in Middle-Aged and Older Adults | Not interested intervention |
| Gertsik | 2012 | Omega-3 Fatty Acid Augmentation of Citalopram Treatment for Patients With Major Depressive Disorder | Duplicate |
| González | 2011 | Omega-3 fatty acids as adjunctive of antidepressant therapy and its effects on brain-derived neurotrophic factor in serum, monocytes and lymphocytes | Not interested outcome |
| Hashimoto | 2015 | Beneficial effects of dietary docosahexaenoic acid intervention on cognitive function and mental health of the oldest elderly in Japanese care facilities and nursing homes | Duplicate |
| Hansen | 2010 | Fish consumption and heart rate variability: Preliminary results | Not interested outcome |
| Huntington Study Group | 2008 | Randomized controlled trial of ethyl-eicosapentaenoic acid in huntington disease | Not interested outcome |
| McGorry | 2016 | Effect of ω-3 Polyunsaturated Fatty Acids in Young People at Ultrahigh Risk for Psychotic Disorders | Duplicate |
| Zanarini | 2003 | Omega-3 Fatty Acid Treatment of Women With Borderline Personality Disorder: A Double-Blind, Placebo-Controlled Pilot Study | Not interested outcome |

**Supplementary Table 4.** Characteristics of the trials included in the meta-analysis of omega-3 fatty acids and anxiety symptoms.

| Author, years, country (ref.) | Participants | Female, n (%) | Age range (mean), years | Anxiety  Scale | Any mood medication usage | Intervention | Comparison | Follow-up duration, weeks | Baseline BMI, kg/m^2^ | Drop out | Baseline anxiety risk |
| --- | --- | --- | --- | --- | --- | --- | --- | --- | --- | --- | --- |
| Antypa, 2012, Netherlands [1] | 71 depressed participants | 58  (81%) | 18-65  (24.6 ± 13.2) | POMS | Yes (citalopram) | 3 g n-3 PUFA/d | Placebo | 4 | I: 21.5  C: 21.6 | 1 | High risk |
| Antypa, 2009, Netherlands [2] | 54 healthy participants | 44  (81%) | (22.4 ± 5.4) | POMS | No | 3 g n-3 PUFA/d | Placebo | 4 | I: 21.9  C: 21.8 | 2 | Low risk |
| Bradbury, 2017, Australia [3] | 90 participants with stress | 64  (71%) | (44.3 ± 13.2) | DASS-anxiety | No | 2.2 g EPA + 0.44 g DHA, daily | Placebo | 12 | I: 25.3  C: 26.4 | I: 7  C: 8 | Low risk |
| Buydens-Branchey, 2008, New York [4] | 22 substance abusers | NR | (51.1 ± 9.8) | Modified POMS | Mixed (antidepressants) | 3 g EPA + DHA, daily | Placebo | 12 | I: 26.1  C: 24.7 | NR | Medium risk |
| Carney, 2020, USA [5] | 144 patients with DSM-V MDD | 56  (38%) | (59.5 ± 13.4) | BAI | Yes  (sertraline) | 2 g of EPA, daily | Placebo | 10 | I: 35.2  C: 35.4 | I: 1  C: 1 | High risk |
| Carney, 2009, USA[6] | 122 patients with MMD & CHD | 41  (34%) | (58.35 ± 12.7) | BAI | Yes  (sertraline) | 930 mg of EPA + 750 mg of DHA, daily | Placebo | 10 | I: 33.8  C: 32.6 | I: 3  C: 4 | High risk |
| Cohen, 2013, USA [7] | 365 healthy participants | 365 (100%) | 40–62  (54.8 ± 5.2) | GAD-7 | No | E-EPA (425 mg), DHA (100 mg), + other omega-3s (90 mg),daily | Placebo | 12 | I: 26.8  C: 27.0 | I: 17  C: 14 | Low risk |
| Dretsch, 2014, Iraq [8] | 106 healthy participants | 17  (16%) | 18–55  (31.4 ± 10.6) | ZAS | No | 2.5 g of EPA + DHA, daily | Placebo | 8.5 | NR | 0 | Medium risk |
| Haberka, 2013, Poland [9] | 52 patients with AMI | 7  (13%) | (58 ± 0.6) | STAI | No | 465 mg EPA + 375 mg DHA, daily | Standard pharmaco-therapy | 4 | NR | 0 | High risk |
| Hallahan, 2007, Ireland [10] | 49 patients with self-harm experience | 32  (65%) | 16–64  (30) | PSS | No | 1.2 g EPA + 0.9 g DHA, daily | Placebo | 12 | I: 24.5  C: 24.5 | I: 3  C: 1 | High risk |
| Jackson, 2012, England [11] | 159 healthy participants | 94  (67%) | 18–35  (22.2 ± 0.9) | DASS**-**anxiety | No | 1 g DHA-rich or EPA-rich, daily | Placebo | 12 | I: 24.0  C: 24.8 | NR | Low risk |
| Jahangard, 2018,  Iran [12] | 50 MDD participants | 16  (68%) | 18 – 65 (42.46) | Anxiety Sensitivity Index-3 | NR | 1000 mg w-3PUFA, daily | Placebo | 12 | I: 25.4  C: 27.2 | 0 | High risk |
| Jiang, 2018, USA [13] | 108 patients with CHF & MDD | 58  (54%) | ≥18  (57.9 ± 22.3) | Spielberger Trait & State Anxiety | Yes  (NR) | 400/200 EPA/DHA, 500 mg, 4 times a day | Placebo | 12 | I: 32.2  C: 33.9 | I, EPA/ DHA: 8  I, high-EPA: 12  C: 8 | High risk |
| Kiecolt-Glaser, 2011,  USA [14] | 68 healthy participants | 30  (44%) | 21-29  (23.6 ± 1.9) | BAI | No | 2085 mg EPA + 348 mg DHA, daily | Placebo | 12 | NR | I: 1 | Low risk |
| Lucas, 2009, Canada [15] | 120 participants with moderate-to-severe PD | 120 (100%) | 40–55  (48.7 ± 5.5) | PGWBS | No | 1.05 g E-EPA/d plus 0.15 g E-DHA, daily | Placebo | 8 | I: 29.5  C: 24.6 | I: 4  C: 10 | High risk |
| Pomponi, 2014,  Italy [16] | 24 individuals with mild to moderate Parkinson’ disease | 11  (92%) | (64.0 ±7.5) | HAM-A | No | 800 mg/d DHA | Placebo | 24 | NR | NR | Medium risk |
| Poppitt, 2015,  New Zealand [17] | 102 patients with ischemic stroke | 30  (29%) | ˃45  (64.5 ± 15.6) | GHQ-28 | NR | 0.7 g DHA + 0.3 g EPA, daily | Placebo | 12 | I: 29.2  C: 28.5 | I: 4  C: 3 | Medium risk |
| Ravi, 2016, Iran [18] | 100 depressed HIV-positive patients | 35  (35%) | 18-65 | HADS | Yes  (NR) | 360 mg EPA + 240 mg DHA, twice a day | Placebo | 8 | NR | I: 4  C: 8 | High risk |
| Robinson, 2018,  USA [19] | 50 PD participants | NR | 15–40 | BPRS | Mixed (antidepressants) | EPA 740 mg + DHA 400 mg, daily | Placebo | 16 | I: 24.6  C: 23.1 | I: 11  C: 12 | High risk |
| Sohrabi, 2012,  Iran [20] | 184 PMS participants | 184 (100%) | 20–45  (31.4 ± 10.6) | VAS | Yes  (NR) | 2 g omega-3, daily | Placebo | 12 | I: 22.0  C: 22.6 | I: 7  C: 8 | Medium risk |
| Tayama, 2018,  Japan [21] | 90 depressed participants | 39  (43%) | (40.4 ± 17.2) | K6 | Yes  (NR) | 558 mg DHA + 1,064 mg EPA, daily | Placebo+ Psycho-education | 12 | NR | I: 5  C: 6 | High risk |
| Van de Rest, 2018, Netherlands [22] | 302 healthy participants | 136  (45%) | ≥65  (69.9 ± 5.9) | HADS | No | 1800 mg EPA+DHA, daily or  400 mg EPA+DHA, daily | Placebo | 26 | I: 26.2  C: 26.5 | I1: 1  I2: 0  C: 3 | Low risk |
| Watanabe, 2018,  Japan [23] | 80 healthy participants | 80  (100%) | 20–59  (30.1 ± 8.4) | HADS | No | 1200 mg of EPA + 600 mg DHA, daily | Placebo | 52 | NR | I: 0  C: 5 | Low risk |
| Abbreviations: ACE, Angiotensin-Converting Enzyme; BMI, body mass index; BIS, Barratt Impulsiveness Scale; BAI, Beck Anxiety Inventory; BPRS, Brief Psychiatric Rating Scale; BD, Bipolar Disorder; BPD, Borderline personality disorder; BIS/BAS, Behavioral Inhibition/Behavioral Activation scale, CGI-S, clinical global impression scale-severity of illness; C, control; DASS, Depression Anxiety and Stress Scales; GHQ-28, 28-item General Health Questionnaire; GAD-7, Generalized Anxiety Disorder-7; HAM-A, Hamilton Anxiety Rating Scale; HADS, Hospital Anxiety and Depression Scale; I, intervention; K6, Kessler Psychological Distress Scale; POMS, Profiles of Mood States; PSS, Perceived Stress Scale; NR, not reported; PGWBS, Psychological General Well-Being Schedule; PSS, Perceived Stress Scale; PUFA, polyunsaturated fatty acids; PD, Psychological distress; STAI, State-Trait Anxiety Inventory; SOFAS, Social and Occupational Functioning Assessment Scale;, SANS, Brief Psychiatric Rating Scale; Scale for the Assessment of Negative Symptoms, , VAS, visual analogue score; YMRS, Young Mania Rating Scale; ZAS, Zung Anxiety Scale. | | | | | | | | | | | |

**Supplementary Table 5.** Risk of bias assessment of trials included in the meta-analysis of omega-3 fatty acids supplementation on anxiety symptoms (ROB2).

| Reference | Bias arising from the randomization process | Bias due to deviations from the intended interventions | Bias due to missing outcome data | Bias in measurement of the outcome | Bias in selection of the reported result | Overall |
| --- | --- | --- | --- | --- | --- | --- |
| Antypa, 2012 | Low risk | Low risk | Low risk | Low risk | Low risk | Low risk |
| Bradbury, 2017 | Low risk | Low risk | Low risk | Low risk | Low risk | Low risk |
| Carney, 2009 | Low risk | Low risk | Low risk | High risk | High risk | High risk |
| Carney, 2020 | Low risk | Some concerns | Low risk | Some concerns | High risk | High risk |
| Cohen, 2014 | Low risk | Low risk | Low risk | Low risk | High risk | High risk |
| Dretsch, 2014 | Low risk | Some concerns | Low risk | Low risk | Some concerns | Some concerns |
| Herberka, 2013 | Low risk | Some concerns | Low risk | Low risk | High risk | High risk |
| HallaHan, 2007 | Low risk | High risk | High risk | Low risk | Low risk | High risk |
| Jackson, 2012 | Some concerns | High risk | High risk | High risk | Low risk | High risk |
| Jahangard, 2018 | Low risk | Some concerns | Low risk | Low risk | Low risk | Some concerns |
| Antypa, 2009 | Low risk | High risk | High risk | Low risk | Low risk | High risk |
| Buydens, 2008 | Low risk | High risk | High risk | Low risk | Low risk | High risk |
| Jiang, 2018 | Low risk | Low risk | Low risk | Low risk | Low risk | Low risk |
| Kiecolt-Glaser, 2011 | Low risk | High risk | Low risk | Low risk | Low risk | High risk |
| Lucas, 2009 | Low risk | Low risk | Low risk | Low risk | Low risk | Low risk |
| Pomponi, 2014 | Low risk | Low risk | Low risk | Low risk | Low risk | Low risk |
| Poppitt, 2015 | Low risk | Some Concerns | Low risk | Low risk | Low risk | Some concerns |
| Ravi, 2016 | Low risk | High risk | High risk | Low risk | Low risk | High risk |
| Robinson, 2018 | Low risk | Low risk | Low risk | Low risk | Low risk | Low risk |
| Sohrabi | Some concerns | High risk | High risk | Low risk | Low risk | High risk |
| Tayama, 2018 | High risk | Low risk | High risk | Low risk | Low risk | High risk |
| van de Rest, 2018 | Low risk | Low risk | Low risk | Low risk | Low risk | Low risk |
| Watanabe, 2018 | Some concerns | Low risk | Low risk | Low risk | Low risk | Some concerns |

**Supplementary Table 6.** GRADE evidence table for the effects of omega-3 fatty acids on primary and secondary outcomes.

| **Certainty assessment** | | | | | | | **№ of patients** | | **Effect** | | **Certainty** | **Importance** |
| --- | --- | --- | --- | --- | --- | --- | --- | --- | --- | --- | --- | --- |
| **№ of studies** | **Study design** | **Risk of bias** | **Inconsistency** | **Indirectness** | **Imprecision** | **Other considerations** | **[intervention]** | **[comparison]** | **Relative (95% CI)** | **Absolute (95% CI)** |  |  |
| **Anxiety symptoms** | | | | | | | | | | | | |
| 23 | randomised trials | serious^a^ | serious^b^ | not serious | serious^c^ | none | 1093 | 1096 | - | SMD **0.7 SD lower** (1.17 lower to 0.22 lower) | ⨁⨁◯◯ Low | IMPORTANT |
| **Adverse event** | | | | | | | | | | | | |
| 8 | randomised trials | not serious | not serious | not serious | serious^d^ | none | 171/574 (29.8%) | 153/587 (26.1%) | **OR 1.20** (0.89 to 1.61) | **37 more per 1,000** (from 22 fewer to 101 more) | ⨁⨁⨁◯ Moderate | IMPORTANT |
| **Emotional well-being** | | | | | | | | | | | | |
| 1 | randomised trials | not serious | serious^e^ | serious^e^ | serious^d^ | none | 36 | 36 | - | SMD **0.23 SD lower** (0.69 lower to 0.23 higher) | ⨁◯◯◯ Very low | IMPORTANT |
| **General health** | | | | | | | | | | | | |
| 1 | randomised trials | not serious | serious^e^ | serious^f^ | serious^d^ | none | 36 | 36 | - | SMD **0.25 SD lower** (0.71 lower to 0.21 higher) | ⨁◯◯◯ Very low | IMPORTANT |
| **Pain** | | | | | | | | | | | | |
| 1 | randomised trials | not serious | serious^e^ | serious^f^ | serious^d^ | none | 36 | 36 | - | SMD **0.33 SD lower** (0.79 lower to 0.13 higher) | ⨁◯◯◯ Very low | IMPORTANT |
| **Physical component scale** | | | | | | | | | | | | |
| 2 | randomised trials | not serious | serious^e^ | serious^g^ | serious^d^ | none | 87 | 87 | - | SMD **0.19 SD lower** (0.48 lower to 0.11 higher) | ⨁◯◯◯ Very low | IMPORTANT |
| **Social functioning** | | | | | | | | | | | | |
| 1 | randomised trials | not serious | serious^e^ | serious^f^ | serious^d^ | none | 36 | 36 | - | SMD **0.52 SD higher** (0.05 higher to 0.98 higher) | ⨁◯◯◯ Very low | IMPORTANT |

**CI:** confidence interval; **OR:** odds ratio; **SMD:** standardized mean difference

#### Explanations

a. Most studies were rated to have serious risk of bias, and there was a significant subgroup difference by risk of bias, where trials with high risk of bias indicated larger effects and those with a low risk of bias showed a non-significant effect. Downgraded.

b. Serious inconsistency since I^2^=97%. Downgraded.

c. The effect size surpassed the minimal clinically important difference, but the upper bound of the 95%CI overlapped the MCID (0.5). Downgraded.

d. Serious imprecision since optimum information size was not met. Downgraded.

e. Serious risk of bias due to incomplete outcome data. Downgraded.

f. Serious indirectness since only one study was available. Downgraded.

g. Serious indirectness since only two studies were available. Downgraded.

**Supplementary Figure 1.** The effects of omega-3 fatty acids (each 1 g/d) on anxiety symptoms.

**Supplementary Figure 2.** Funnel plot of the effects of omega-3 fatty acids (each 1 g/d) on anxiety symptoms.

**Supplementary Figure 3.** The effects of omega-3 fatty acids (each 1 g/d) on adverse event.

**Supplementary Figure 4.** The effects of omega-3 fatty acids (each 1 g/d) on adverse events.

**References**

1. Antypa N, Smelt AH, Strengholt A, Van der Does AJ: **Effects of omega-3 fatty acid supplementation on mood and emotional information processing in recovered depressed individuals**. *J Psychopharmacol* 2012, **26**(5):738-743.

2. Antypa N, Van der Does AJ, Smelt AH, Rogers RD: **Omega-3 fatty acids (fish-oil) and depression-related cognition in healthy volunteers**. *J Psychopharmacol* 2009, **23**(7):831-840.

3. Bradbury J, Myers SP, Meyer B, Brooks L, Peake J, Sinclair AJ, Stough C: **Chronic Psychological Stress Was Not Ameliorated by Omega-3 Eicosapentaenoic Acid (EPA)**. *Front Pharmacol* 2017, **8**:551.

4. Buydens-Branchey L, Branchey M, Hibbeln JR: **Associations between increases in plasma n-3 polyunsaturated fatty acids following supplementation and decreases in anger and anxiety in substance abusers**. *Prog Neuropsychopharmacol Biol Psychiatry* 2008, **32**(2):568-575.

5. Carney RM, Freedland KE, Rubin EH, Rich MW, Steinmeyer BC, Harris WS: **A Randomized Placebo-Controlled Trial of Omega-3 and Sertraline in Depressed Patients With or at Risk for Coronary Heart Disease**. *J Clin Psychiatry* 2019, **80**(4).

6. Carney RM, Freedland KE, Rubin EH, Rich MW, Steinmeyer BC, Harris WS: **Omega-3 augmentation of sertraline in treatment of depression in patients with coronary heart disease: a randomized controlled trial**. *Jama* 2009, **302**(15):1651-1657.

7. Cohen LS, Joffe H, Guthrie KA, Ensrud KE, Freeman M, Carpenter JS, Learman LA, Newton KM, Reed SD, Manson JE *et al*: **Efficacy of omega-3 for vasomotor symptoms treatment: a randomized controlled trial**. *Menopause* 2014, **21**(4):347-354.

8. Dretsch MN, Johnston D, Bradley RS, MacRae H, Deuster PA, Harris WS: **Effects of omega-3 fatty acid supplementation on neurocognitive functioning and mood in deployed U.S. soldiers: a pilot study**. *Mil Med* 2014, **179**(4):396-403.

9. Haberka M, Mizia-Stec K, Mizia M, Gieszczyk K, Chmiel A, Sitnik-Warchulska K, Gąsior Z: **Effects of n-3 polyunsaturated fatty acids on depressive symptoms, anxiety and emotional state in patients with acute myocardial infarction**. *Pharmacol Rep* 2013, **65**(1):59-68.

10. Hallahan B, Hibbeln JR, Davis JM, Garland MR: **Omega-3 fatty acid supplementation in patients with recurrent self-harm. Single-centre double-blind randomised controlled trial**. *Br J Psychiatry* 2007, **190**:118-122.

11. Jackson PA, Deary ME, Reay JL, Scholey AB, Kennedy DO: **No effect of 12 weeks' supplementation with 1 g DHA-rich or EPA-rich fish oil on cognitive function or mood in healthy young adults aged 18-35 years**. *Br J Nutr* 2012, **107**(8):1232-1243.

12. Jahangard L, Sadeghi A, Ahmadpanah M, Holsboer-Trachsler E, Sadeghi Bahmani D, Haghighi M, Brand S: **Influence of adjuvant omega-3-polyunsaturated fatty acids on depression, sleep, and emotion regulation among outpatients with major depressive disorders - Results from a double-blind, randomized and placebo-controlled clinical trial**. *J Psychiatr Res* 2018, **107**:48-56.

13. Jiang W, Whellan DJ, Adams KF, Babyak MA, Boyle SH, Wilson JL, Patel CB, Rogers JG, Harris WS, O'Connor CM: **Long-Chain Omega-3 Fatty Acid Supplements in Depressed Heart Failure Patients: Results of the OCEAN Trial**. *JACC Heart Fail* 2018, **6**(10):833-843.

14. Kiecolt-Glaser JK, Belury MA, Andridge R, Malarkey WB, Glaser R: **Omega-3 supplementation lowers inflammation and anxiety in medical students: a randomized controlled trial**. *Brain Behav Immun* 2011, **25**(8):1725-1734.

15. Lucas M, Asselin G, Mérette C, Poulin MJ, Dodin S: **Ethyl-eicosapentaenoic acid for the treatment of psychological distress and depressive symptoms in middle-aged women: a double-blind, placebo-controlled, randomized clinical trial**. *Am J Clin Nutr* 2009, **89**(2):641-651.

16. Pomponi M, Loria G, Salvati S, Di Biase A, Conte G, Villella C, Righino E, Ciciarelli C, Bria P, La Torre G *et al*: **DHA effects in Parkinson disease depression**. *Basal Ganglia* 2014, **4**(2):61-66.

17. Poppitt SD, Howe CA, Lithander FE, Silvers KM, Lin RB, Croft J, Ratnasabapathy Y, Gibson RA, Anderson CS: **Effects of moderate-dose omega-3 fish oil on cardiovascular risk factors and mood after ischemic stroke: a randomized, controlled trial**. *Stroke* 2009, **40**(11):3485-3492.

18. Ravi S, Khalili H, Abbasian L, Arbabi M, Ghaeli P: **Effect of Omega-3 Fatty Acids on Depressive Symptoms in HIV-Positive Individuals: A Randomized, Placebo-Controlled Clinical Trial**. *Ann Pharmacother* 2016, **50**(10):797-807.

19. Robinson DG, Gallego JA, John M, Hanna LA, Zhang JP, Birnbaum ML, Greenberg J, Naraine M, Peters BD, McNamara RK *et al*: **A potential role for adjunctive omega-3 polyunsaturated fatty acids for depression and anxiety symptoms in recent onset psychosis: Results from a 16 week randomized placebo-controlled trial for participants concurrently treated with risperidone**. *Schizophr Res* 2019, **204**:295-303.

20. Sohrabi N, Kashanian M, Ghafoori SS, Malakouti SK: **Evaluation of the effect of omega-3 fatty acids in the treatment of premenstrual syndrome: "a pilot trial"**. *Complement Ther Med* 2013, **21**(3):141-146.

21. Tayama J, Ogawa S, Nakaya N, Sone T, Hamaguchi T, Takeoka A, Hamazaki K, Okamura H, Yajima J, Kobayashi M *et al*: **Omega-3 polyunsaturated fatty acids and psychological intervention for workers with mild to moderate depression: A double-blind randomized controlled trial**. *J Affect Disord* 2019, **245**:364-370.

22. van de Rest O, Geleijnse JM, Kok FJ, van Staveren WA, Hoefnagels WH, Beekman AT, de Groot LC: **Effect of fish-oil supplementation on mental well-being in older subjects: a randomized, double-blind, placebo-controlled trial**. *Am J Clin Nutr* 2008, **88**(3):706-713.

23. Watanabe N, Matsuoka Y, Kumachi M, Hamazaki K, Horikoshi M, Furukawa TA: **Omega-3 fatty acids for a better mental state in working populations - Happy Nurse Project: A 52-week randomized controlled trial**. *J Psychiatr Res* 2018, **102**:72-80.
